# The Nuclear Lamina as an Organizer of Chromosome Architecture

**DOI:** 10.3390/cells8020136

**Published:** 2019-02-08

**Authors:** Yuri Y. Shevelyov, Sergey V. Ulianov

**Affiliations:** 1 Department of Molecular Genetics of Cell, Institute of Molecular Genetics, Russian Academy of Sciences, Moscow 123182, Russia; shevelev@img.ras.ru; 2 Division of the Regulation of Transcription and Chromatin Dynamics, Institute of Gene Biology, Russian Academy of Sciences, Moscow 119334, Russia; sergey.v.ulyanov@gmail.com

**Keywords:** nuclear lamina, nuclear periphery, nuclear envelope, lamin, LAD, TAD, heterochromatin, HP1, H3K9me2/3

## Abstract

The nuclear lamina (NL) is a meshwork of lamins and lamin-associated proteins adjoining the inner side of the nuclear envelope. In early embryonic cells, the NL mainly suppresses background transcription, whereas, in differentiated cell types, its disruption affects gene expression more severely. Normally, the NL serves as a backbone for multiple chromatin anchoring sites, thus shaping the spatial organization of chromosomes in the interphase nucleus. However, upon cell senescence, aging, or in some types of terminally differentiated cells and lamin-associated diseases, the loss of NL-chromatin tethering causes drastic alterations in chromosome architecture. Here, we provide an overview of the recent advances in the field of NL-chromatin interactions, focusing on their impact on chromatin positioning, compaction, repression, and spatial organization.

## 1. Introduction

In eukaryotes, the densely packed heterochromatin is mainly located at the nuclear periphery, whereas the less compact euchromatin occupies a more interior nuclear position. Heterochromatin is subdivided into densely-packed constitutive heterochromatin, covering pericentromeric and telomeric chromosomal regions, and less condensed facultative heterochromatin located in the chromosomal arms. Recent electron microscopy observations indicate that during interphase chromatin is represented by a 5- to 24-nm nucleosomal chain aggregated into irregular clusters with a higher packaging density at the nuclear periphery [1]. The nuclear lamina (NL) is a meshwork consisting of A- and B-type lamins and lamin-associated proteins which lines the inner nuclear membrane [2] and participates in the compaction of peripheral chromatin. Chromosomal regions interacting with the NL, the so-called lamina-associated domains (LADs), have been identified in various organisms from nematode to human using either DNA adenine methyltransferase identification (DamID) [3] or chromatin immunoprecipitation (ChIP) techniques [4,5,6,7,8,9,10]. LADs appear to contain mostly silent or weakly-expressed genes [4,6], thus supporting the idea that NL is a repressive nuclear compartment. As the ChIP approach captures mostly stable interactions, whereas DamID also detects the transient contacts, it became clear that LADs are not only in contact with but are attached to the NL. However, the influence of NL-chromatin interactions on the overall chromosome architecture and gene expression is still the subject of ongoing research.

## 2. Mechanisms of LADs Tethering to the NL

Several studies examined which components of the NL might tether chromatin. Usually, the positions of endogenous loci or *lacO*-tagged genomic regions relative to the nuclear envelope (NE) were assayed by fluorescence in situ hybridization (FISH) or by the fluorescence imaging of LacI-GFP upon depletion of candidate proteins. The loss of tethering is expected to result in the removal of loci from the NE. In such a way, histone deacetylase HDAC3 (even lacking its catalytic activity) in the complex with the NE transmembrane (NET) protein Lap2β and the DNA-binding protein cKrox was shown to participate in LAD tethering to the NE in mice cells [11,12]. Similarly, HDAC3 appears to be required for the retention of a LAD at the nuclear periphery in *Drosophila* S2 cells [13]. Furthermore, depletion of PRR14 protein, which associates with the A-type lamin at the NL and binds heterochromatin protein 1α (HP1α), resulted in the redistribution of histone H3 lysine 9 di/trimethylated (H3K9me2/3) chromatin from the nuclear periphery to the nuclear interior in human cells [14]. In a similar approach, the CEC-4 component of the NL was revealed as a direct tether of H3K9me2/3-marked chromatin in nematodes [15].

Lamin-B-receptor (LBR), the NET protein associated with the B-type lamin, is one of the participants which maintain the peripheral position of heterochromatin during the early embryonic development of mammals [16]. LBR and lamins interact with the same genome regions as revealed by DamID [17]. LBR forms a complex with HP1α [18,19] and thus can link the H3K9me2/3-modified chromatin of LADs [4,20] as well as pericentromeric regions to the NL. LBR also binds the histone H4 lysine 20 dimethylated (H4K20me2) mark, which is abundantly represented at the nuclear periphery [21]. The naturally-occurring down-regulation of LBR in mouse olfactory sensory neurons results in the aggregation of pericentromeric heterochromatin into foci located far from the NL, whereas an ectopic LBR expression leads to the shift of these foci toward the nuclear periphery [22]. Depletion of LBR in two human cancer cell lines also results in the relocalization of pericentromeric heterochromatin from the NL to the nucleoplasm [23], thus illuminating its chromatin tethering function. Apart from LBR, which is most important in early development, several tissue-specifically expressed NET proteins were shown to tether particular loci or even whole chromosomes to the NE, specifically in differentiated mammalian cells [24,25].

Lamins themselves might participate in chromatin tethering based on their ability to bind DNA, histones, and chromatin in in vitro assays [26,27,28]. In *Drosophila*, B-type lamin is required for in vivo chromatin tethering as its disruption results in the relocalization of particular gene loci from the nuclear periphery both in embryonic S2 cells and in neuroblasts [29,30]. Moreover, knock-out of the *Lmnb1* gene in mouse embryonic fibroblasts results in the relocation of chromosome 18 to the nuclear interior [31]. Similarly, knock-out of the *Lmna* gene in mouse postmitotic cells lacking LBR expression leads, in some cell types, to the so-called “inverted” nuclear architecture [32], characterized by heterochromatin aggregation in the center of nucleus and euchromatin facing the NE [16]. Finally, upon depletion of B-type lamin in S2 cells (which also lack the A-type lamin), not only particular loci but a bulk chromatin mass is detached from the NE and shifted towards the nuclear interior [33]. However, upon loss of all lamins, general chromatin detachment from the NL was not observed in mouse embryonic stem cells (mESCs) [34]. Under these conditions, facultative LADs were detached, while the constitutive LADs were retained at the nuclear periphery [34,35]. Although it seems likely, it is not yet proven that lamins tether chromatin directly, as their absence leads to the mislocalization of many other components of NL as well as of nuclear pore complexes [36,37,38,39].

What might be the reasons for the different chromatin responses to the loss of all lamins in embryonic cells of *Drosophila* and mammals? In contrast to mammals, where the presence of either LBR or lamin A/C is necessary to keep heterochromatin at the nuclear periphery [16], the depletion of LBR and simultaneous absence of A-type lamin in *Drosophila* S2 cells did not lead to the notable alteration of chromatin position relative to the NE [33]. Therefore, in mESCs the loss of all lamins may not be sufficient to completely detach chromatin from the NE [40,41].

**Figure 1:**
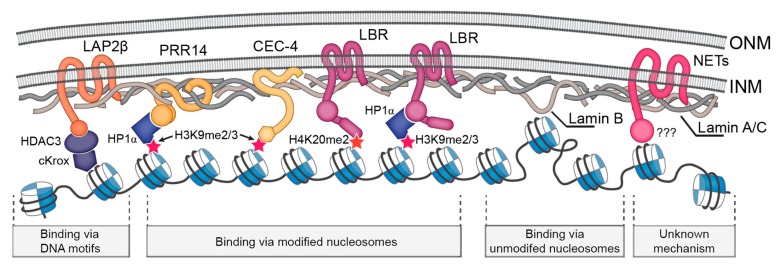
Schematic representation of the main NL-chromatin tethering mechanisms.

Three types of NL-chromatin tethering mechanisms are summarized in Figure 1.

Notably, the results of the aforementioned experiments show that, upon loss of tethering components, chromatin occupies a more interior position in the nucleus. This clearly indicates that the attachment of interphase chromosomes to the NE slightly stretches them. Ulianov et al. [33] proposed that macromolecular crowding [42] and inter-nucleosomal interactions within the topologically associating domains (TADs) [43,44,45,46] result in a slight chromosome contraction upon loss of their tethering to the NL.

## 3. Impact of the NL on LADs Compaction and Repression

It is well-established that LADs mainly contain genes which are weakly-expressed or silent [4,6]. Several findings in mammals and *Drosophila* indicate that the bodies of expressed genes may still be located within LADs, yet their promoters most likely lose contact with the NL [5,47,48,49]. Therefore, the NL is an unfavorable environment for transcription. Furthermore, artificial tethering of weakly-expressed reporters to the NL results in their silencing [50,51,52,53], thus indicating that the NL has the capacity to establish gene repression. However, judging from single-cell DamID analysis, less than one third of all LADs revealed in a cell population are localized to the NL in individual cells, with the NL-attached regions being stochastically reshuffled after each mitosis [54,55]. These results indicate that if the NL strongly suppresses gene transcription, different genes should be silenced in various cells of the same lineage, which seems highly unlikely. Although genes in the NL-untethered LADs may be repressed due to their interaction with other repressive nuclear compartments, such as nucleoli [54] or chromocenters, LADs appear to overlap only weakly with the nucleoli-associated domains [56] or, in mESCs, with the pericentromere-associated domains [57]. Furthermore, lamins appear to be dispensable in some mouse cell types including mESCs [40,41]. Likewise, loss of CEC-4 tethering in nematode shifted heterochromatin from the nuclear periphery but did not lead to gene activation [15]. Therefore, it still remains unclear whether genes in LADs are silent due to their contact with the NL, or whether they are repressed by NL-independent mechanism(s) and, because of this, repositioned to the NL. In other words, the question exists how the NL impacts on the compaction and repression of chromatin in LADs.

Two recent studies addressed this question by employing the technique of chromosome conformation capture with high throughput sequencing (Hi-C, [58]) on mESCs or embryonic *Drosophila* S2 cells lacking all lamins [33,34]. Strikingly, in both mouse and *Drosophila* cells, a fraction of NL-attached TADs became less compact upon the loss of all lamins [33,34]. The decompaction of these TADs in *Drosophila* S2 cells was accompanied by an increased level of histone H3 acetylation and background transcription in these regions [33]. Given that a fraction of HDACs was revealed in the mammalian NL proteome [59,60], Ulianov et al. [33] suggest that HDACs linked to the NL may additionally deacetylate histones in LADs. Furthermore, polymer simulations indicate that the attachment of LADs to the NL may mechanically compact their chromatin [33]. Zheng et al. [34] hypothesize that LADs at the NL may become more densely packed due to the lamin meshwork which wraps around them and restricts chromatin mobility. Collectively, these findings strongly support the concept that the NL enhances the compaction and repression of chromatin in LADs. Yet, it should be noted that the impact of the NL on LAD compaction is rather weak, at least in embryonic cells. Ulianov et al. [33] suggest that chromatin deacetylation and compaction mediated by the NL are mainly directed to suppress the occasional binding of transcription factors in LADs in order to diminish the background transcription in these regions.

Interestingly, RNA-seq analysis indicates that a loss of all lamins does not only up-regulate the background transcription in LADs but also alters the expression of genes positioned in the nucleoplasm [33,34,40]. One of the explanations is that upon NL disruption, chromatin interactions may be changed also in the nuclear interior. Previously, using Hi-C, it was found that active and inactive chromatin in mammalian cells is spatially segregated into A and B compartments [58]. Importantly, in both mouse and *Drosophila*, chromatin compartmentalization was impaired upon lamin loss [33,34]. Therefore, this gain and loss of inter-domain interactions might affect gene expression in the nuclear interior. Additionally, lamin A/C which is expressed later in development and can interact not only with the inactive chromatin at the NL but also with the active chromatin in the nucleoplasm of mammalian cells [61,62], may affect the expression of genes located in the inter-LAD regions. This latter effect may explain the variety of human diseases, collectively named laminopathies, which are mainly caused by the mutations in the *LMNA* gene [63].

## 4. Heterochromatin Maturation upon Cell Differentiation

In mammals, the transition from undifferentiated or pluripotent to a more differentiated cell state is accompanied by an increase in the packaging density of peripheral chromatin [64], and a similar picture is seen in nematodes [65]. However, what happens with the peripheral chromatin upon cell differentiation in *Drosophila* remains mostly unexplored, not least because in this model organism, LADs have previously been mapped only in the Kc167 cell line of embryonic origin [6]. Recently, this knowledge gap was filled, and LADs as well as HP1a- and Polycomb (Pc)-associated domains were identified in various organs/cell types of *Drosophila* third instar larvae, including the fat body, brain, glia, and neurons [49,66]. In contrast to mammals, where LADs are enriched with H3K9me2 mark along their length as well as with histone H3 lysine 27 trimethylated (H3K27me3) mark at their borders [4,20], LADs in *Drosophila* Kc167 cells paradoxically do not contain any H3K9me2/HP1a, while ~40% of their length is covered by Pc [6,67]. Strikingly, in *Drosophila* neurons and, to a lesser degree, in glia and fat bodies, LADs appear to be widely covered by HP1a both in chromosome arms and in the pericentromeric regions [49]. Moreover, centromeres, embedded in the constitutive heterochromatin, are localized closer to the NE in neurons than in Kc167 cells [49]. At the same time, Pc-enriched LADs are rather conservative among different *Drosophila* cell types [49]. Therefore, peripheral heterochromatin possesses a similar composition in mammals and in differentiated *Drosophila* cells.

Several studies indicate that in differentiated mammalian cells, the disruption of NL components exerts a stronger effect on gene expression than in mESCs (for example, [25,68]). In differentiated *Drosophila* cells, genes residing in LADs, which are bound with HP1a or Pc, are expressed more weakly than genes residing in LADs without HP1a and Pc binding [49]. Moreover, upon aging, the level of B-type lamin is prominently reduced in the major immune organ in *Drosophila*, the fat body, which causes the HP1a/H3K9me2 loss and de-repression of a set of immune response genes [69]. It is supposed that when cell lineages are committed for terminal differentiation, the maturated heterochromatin imposes an additional level of repression on genes that may interfere with proper differentiation, thus ensuring their irreversible and effective silencing [49,70,71]; however, this repression is weakened upon aging. It is still to be determined whether NL disruption would lead to more severe gene expression defects in terminally differentiated *Drosophila* cells than in embryonic cells. Interestingly, while in various mammalian tissues heterochromatin, maturation is associated with the expansion of Pc/H3K27me3 domains [70], in *Drosophila* neurons, it mainly relies on the genome-wide spreading of HP1a [49,72].

In differentiated cell types, the HP1a occupancy in LADs may be mediated by its capacity to form liquid droplets resulting in the phase separation of heterochromatin and euchromatin [73,74]. As the efficiency of phase separation, among others, may be determined by the kinetics of HP1a binding to its targets [75], in the postmitotic terminally differentiated cells, such as neurons, HP1a may have sufficient time to form liquid droplets on the transposable element (TE) insertion sites, abundant in LADs and then to spread on the adjacent chromatin [49]. In support of this idea, it was found that the postmitotic state, but not the differentiation per se, is necessary for accumulation of heterochromatin in terminally differentiated cells [76]. Moreover, a spreading of H3K9me2-modified heterochromatin from the TEs on nearby DNA was found to occur at distances of up to 20-kb in *Drosophila* [77]. Finally, the concentration of HP1a in the nucleus may also be an important parameter for HP1a spreading along chromosome arms [75].

## 5. Loss of NL-Chromatin Tethering Is Linked to Drastic Alterations of Chromosome Architecture

**Figure 2:**
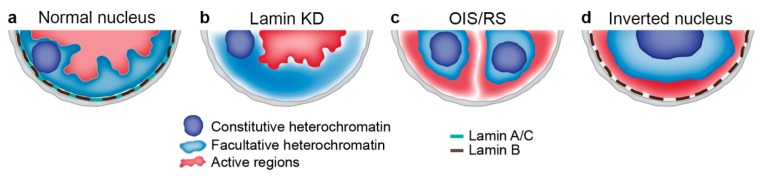
Schematic representation of different types of chromosome architecture generated upon loss of NL-chromatin tethering. (**a**) Conventional nuclear architecture in most mammalian cell types. (**b**) Nuclear architecture in *Drosophila* S2 cells lacking both A- and B-type lamins. (**c**) Nuclear architecture upon OIS or RS. (**d**) “Inverted” nuclear architecture in rod photoreceptors.

Cellular senescence is an irreversible arrest of the cell cycle in response to stress induced by activated oncogenes, DNA damage, oxidants, or telomere shortening [78]. A common characteristic is the formation of senescence-associated heterochromatin foci (SAHF) [79] which represent the condensed individual chromosome territories. These consist of the H3K9me3-enriched central cores with the outer layers containing H3K27me3-marked chromatin (Figure 2) [80,81]. Remarkably, the oncogene-induced senescence (OIS) is associated with the down-regulation of A- and B-type lamins as well as of some NETs including LBR [82,83], implying that in these cells the main heterochromatin tethers are disrupted. Similarly, a decline in the level of B-type lamin was revealed during normal aging in both humans and *Drosophila* [69,84].

The question then arises whether the absence of lamins is the cause or the consequence of senescence. In fact, depletion of lamin B1 induces premature senescence and SAHF formation in some human cell lines [9,10,82,83], pointing to a causative role for lamin B1 but not in others [84]. Regardless, the detachment of heterochromatin from the NL seems to be an important prerequisite for SAHF formation. Indeed, according to ChIP or DamID with lamin B1, NL-chromatin interactions appear to be drastically reduced in OIS cells compared to cycling cells, with the H3K9me2/3-enriched constitutive LADs being especially affected [9,85]. At the same time, regions carrying H3K27me3 mark are partially retained at the nuclear periphery upon OIS [9,85]. Strikingly, according to DamID [85], but not to ChIP-seq [9], chromosomal regions containing actively expressed genes begin to interact with the remains of the NL upon OIS (Figure 2), thus indicating that the transient contacts but not the attachment of active chromatin to disrupted NL is enhanced in OIS cells. Hi-C analysis of the three-dimensional chromatin organization reveals the preservation of the genome-wide TAD profile upon OIS [86]. Remarkably, consistent with the aggregation of H3K9me3-marked heterochromatin in SAHF, distant interactions between TADs that were attached to the NL and enriched with H3K9me3 in cycling cells are increased upon OIS [86]. The interactions within TADs are altered depending on their NL-attachment status in cycling cells: upon OIS, the interactions within the NL-attached TADs are reduced, while they are enhanced within other TADs [86]. These findings indicate that NL-attached TADs become less compact after they have lost interactions with the NL.

Interestingly, FISH analysis performed on cells undergoing replicative senescence (RS), which also lack peripheral heterochromatin [87] but infrequently form SAHF, indicates that whole chromosome territories become more compact in RS cells [88]. In agreement with the general compaction of chromosomes, Hi-C interactions in RS cells appear to be uniformly increased within TADs, without any dependence on chromatin state or NL-attachment [88]. However, long-range Hi-C interactions are decreased relative to the cycling cells [88]. Criscione et al. [88] suggest that the general chromatin condensation observed in RS cells may be attributed to the long-lasting maturation of the RS phenotype (months) as compared to the short maturation time during OIS (days).

Another example of strong alterations of chromosome architecture comes from the studies of Hutchinson-Gilford progeria syndrome (HGPS) which is caused by the mutated form of lamin A [89]. Microscopy observations indicate that in HGPS fibroblasts, tethering of heterochromatin to the NL is abolished [90]. Nevertheless, HGPS cells do not form SAHF-like structures, likely because they contain a reduced level of H3K9me3 [91]. In agreement with the loss of peripheral heterochromatin, H3K27me3 methylation in the regions losing lamin A/C binding is also diminished in HGPS cells [92]. In contrast, at the same time some of the gene-rich regions gain both the H3K27me3 mark and lamin A/C binding [92]. Hi-C analysis demonstrates a profound decline in long-range interactions at later passages of HGPS cells associated with premature senescence [92]. This resembles the RS cells [88] although with a higher degree of compartmentalization loss.

One of the most radical changes in chromosome architecture was described in the mature rod photoreceptor cells of animals with nocturnal vision [32]. Their “inverted” nuclear organization is characterized by the coalescence of constitutive heterochromatin in the single chromocenter in the middle of nucleus, with the concentric layer of facultative heterochromatin around it and a thin euchromatin layer adjoining the NE (Figure 2) [32,93]. Such an organization is mediated by the loss of both LBR and lamin A/C, which is sufficient for chromatin detachment from the NE [16]. Although chromatin in rod photoreceptor cells is noticeably depleted of the “open”, accessible configuration [94,95], B-type lamins as well as many other genes are actively expressed in these cells [32,95]. Positioning of euchromatin at the nuclear periphery raises the question whether these cells contain LADs consisting of active chromatin. This seems likely since a fraction of LADs in OIS cells contain actively expressed genes [85]. It was intriguing to find out what happens with the chromosome architecture at the TAD level in this specific type of terminally differentiated cells. Surprisingly, Hi-C analysis has shown that TADs were unaffected by the inversion [96]. Moreover, chromatin compartmentalization was also not impaired in rod photoreceptors compared to cells with the conventional architecture [96]. Based on polymer modelling, the authors propose that the loss of anchorage of heterochromatin at the NL along with the ability to aggregate of different heterochromatin regions - drives the inverted nuclear organization, whereas the attachment of heterochromatin to the NL is required for the maintenance of conventional nuclear architecture [96].

## 6. Conclusions

We assume the existence of at least three types of NL-chromatin tethering mechanisms. NL components may bind with either modified (1) or unmodified (2) nucleosomes or with the specific DNA motifs (3) present in LADs (Figure 1). As LADs in mammals and nematode contain H3K9me2/3-modified nucleosomes along their length, the components of the NL which bind this mark, such as LBR/HP1α [18,97,98] or PRR14/HP1α [11,12] in mammals and CEC-4 [15] in nematode, maintain LAD attachment to the NE. In postmitotic *Drosophila* neurons, LADs bound by HP1a [49] might hypothetically be attached to the NE with the involvement of the same mechanism. In mammals, the YY1 protein interacting with the H3K27me3 mark, which is enriched at LAD borders, also participates in LAD tethering [99]. These types of interactions represent NL-chromatin tethering mechanism of the first type. Another potential mechanism may rely on the unspecific binding of lamins with the non-modified nucleosomes in LADs [33]. It is exemplified by a fraction of LADs in *Drosophila* Kc167 and, likely, S2 cells which are devoid of acetylation as well as of H3K9 and H3K27 methylation [67,100]. Finally, sequence-specific recognition of (GA)n motifs by the cKrox/HDAC3/Lap2β complex in mammals [11] represents an example of the third type of NL-chromatin interactions. Chromatin tethering to the NL via HDAC3 also operates in *Drosophila* S2 cells [13]. Three types of tethering may be redundant with the varying impact of each one in different cell types or organisms.

The effect of NL disruption on gene expression appears to be rather weak, at least in the early embryonic cells [33,40]. It was suggested that in *Drosophila* S2 cells, NL compacts and deacetylates LADs mainly in order to suppress the background transcription within them [33]. However, in differentiated cell types, the lack of specific NL components affects gene expression more severely [25,68,101], although, in case of loss of the A-type lamin, it is not clear to what extent it happens at the NL or in the nuclear interior.

Upon chromatin release from NL-tethering, several types of chromosome architecture were described: for example, removal of total chromatin from the NE with its slight shrinkage and LAD decompaction in *Drosophila* [33]; decompaction of a fraction of LADs in mESCs [34]; heterochromatin redistribution, formation of SAHF, and LAD decompaction upon OIS in mammals [79,85,86]; peripheral heterochromatin loss and general chromosome compaction without SAHF formation upon RS in mammals [87,88]; peripheral heterochromatin loss not accompanied by SAHF formation in HPGS cells [92]; and “inverted” nuclear architecture in mouse rod photoreceptors [32] (Figure 2).

What might be the reason(s) for such variation in nuclear organization triggered by the same cause? Obviously, loss of NL-tethering is only one of the factors that affect the formation of non-conventional architecture. The duration of the post-mitotic phase as well as heterochromatin composition (in particular, its HP1/H3K9me2/3-enrichment) may also be responsible, at least in part, for the diverse outputs. Heterochromatin/euchromatin phase separation mediated by HP1/H3K9me2/3 interactions [73,74,97,98] likely determines the aggregation of LADs into several SAHF in OIS cells or into a single, central heterochromatin focus in rod photoreceptor cells. In *Drosophila* S2 cells, LADs in chromosome arms are almost completely devoid of this type of heterochromatin [67,100]. Moreover, in the late-passage HGPS cells, the H3K9me3 mark is reduced [91] and SAHF-like structures are rarely detected. Yet, some other factors should be invoked to model the peculiarities of chromosome organization upon loss of NL-tethering. Nevertheless, we now know that detachment of chromatin from the NE, at least in some cases, results in its overall contraction [33,88], while the released TADs become decompacted [33,34,86]. Altogether, these studies provide clear evidence that NL-chromatin interactions play a key role in the maintenance of the conventional nuclear architecture, while their disturbances, occurring upon cell senescence, aging, in rare cases of normal differentiation, or in lamin-associated diseases, cause strong alterations of chromatin organization in interphase nucleus.

## References

[1] Ou H.D., Phan S., Deerinck T.J., Thor A., Ellisman M.H., O’Shea C.C. (2017). ChromEMT: Visualizing 3D chromatin structure and compaction in interphase and mitotic cells. Science.

[2] Gruenbaum Y., Foisner R. (2015). Lamins: Nuclear intermediate filament proteins with fundamental functions in nuclear mechanics and genome regulation. Annu. Rev. Biochem..

[3] Van Steensel B., Henikoff S. (2000). Identification of in vivo DNA targets of chromatin proteins using tethered dam methyltransferase. Nat. Biotechnol..

[4] Guelen L., Pagie L., Brasset E., Meuleman W., Faza M.B., Talhout W., Eussen B.H., de Klein A., Wessels L., de Laat W. (2008). Domain organization of human chromosomes revealed by mapping of nuclear lamina interactions. Nature.

[5] Peric-Hupkes D., Meuleman W., Pagie L., Bruggeman S.W., Solovei I., Brugman W., Gräf S., Flicek P., Kerkhoven R.M., van Lohuizen M. (2010). Molecular maps of the reorganization of genome-nuclear lamina interactions during differentiation. Mol. Cell.

[6] Van Bemmel J.G., Pagie L., Braunschweig U., Brugman W., Meuleman W., Kerkhoven R.M., van Steensel B. (2010). The insulator protein SU(HW) fine-tunes nuclear lamina interactions of the *Drosophila* genome. PLoS ONE.

[7] Ikegami K., Egelhofer T.A., Strome S., Lieb J.D. (2010). *Caenorhabditis elegans* chromosome arms are anchored to the nuclear membrane via discontinuous association with LEM-2. Genome Biol..

[8] Lund E., Oldenburg A.R., Delbarre E., Freberg C.T., Duband-Goulet I., Eskeland R., Buendia B., Collas P. (2013). Lamin A/C-promoter interactions specify chromatin state-dependent transcription outcomes. Genome Res..

[9] Sadaie M., Salama R., Carroll T., Tomimatsu K., Chandra T., Young A.R., Narita M., Pérez-Mancera P.A., Bennett D.C., Chong H. (2013). Redistribution of the Lamin B1 genomic binding profile affects rearrangement of heterochromatic domains and SAHF formation during senescence. Genes Dev..

[10] Shah P.P., Donahue G., Otte G.L., Capell B.C., Nelson D.M., Cao K., Aggarwala V., Cruickshanks H.A., Rai T.S., McBryan T. (2013). Lamin B1 depletion in senescent cells triggers large-scale changes in gene expression and the chromatin landscape. Genes Dev..

[11] Zullo J.M., Demarco I.A., Piqué-Regi R., Gaffney D.J., Epstein C.B., Spooner C.J., Luperchio T.R., Bernstein B.E., Pritchard J.K., Reddy K.L. (2012). DNA sequence-dependent compartmentalization and silencing of chromatin at the nuclear lamina. Cell.

[12] Poleshko A., Shah P.P., Gupta M., Babu A., Morley M.P., Manderfield L.J., Ifkovits J.L., Calderon D., Aghajanian H., Sierra-Pagán J.E. (2017). Genome-nuclear lamina interactions regulate cardiac stem cell lineage restriction. Cell.

[13] Milon B.C., Cheng H., Tselebrovsky M.V., Lavrov S.A., Nenasheva V.V., Mikhaleva E.A., Shevelyov Y.Y., Nurminsky D.I. (2012). Role of histone deacetylases in gene regulation at nuclear lamina. PLoS ONE.

[14] Poleshko A., Mansfield K.M., Burlingame C.C., Andrake M.D., Shah N.R., Katz R.A. (2013). The human protein PRR14 tethers heterochromatin to the nuclear lamina during interphase and mitotic exit. Cell Rep..

[15] Gonzalez-Sandoval A., Towbin B.D., Kalck V., Cabianca D.S., Gaidatzis D., Hauer M.H., Geng L., Wang L., Yang T., Wang X. (2015). Perinuclear anchoring of H3K9-methylated chromatin stabilizes induced cell fate in *C. elegans* Embryos. Cell.

[16] Solovei I., Wang A.S., Thanisch K., Schmidt C.S., Krebs S., Zwerger M., Cohen T.V., Devys D., Foisner R., Peichl L. (2013). LBR and lamin A/C sequentially tether peripheral heterochromatin and inversely regulate differentiation. Cell.

[17] Ibarra A., Benner C., Tyagi S., Cool J., Hetzer M.W. (2016). Nucleoporin-mediated regulation of cell identity genes. Genes Dev..

[18] Ye Q., Worman H.J. (1996). Interaction between an integral protein of the nuclear envelope inner membrane and human chromodomain proteins homologous to *Drosophila* HP1. J. Biol. Chem..

[19] Polioudaki H., Kourmouli N., Drosou V., Bakou A., Theodoropoulos P.A., Singh P.B., Giannakouros T., Georgatos S.D. (2001). Histones H3/H4 form a tight complex with the inner nuclear membrane protein LBR and heterochromatin protein 1. EMBO Rep..

[20] Wen B., Wu H., Shinkai Y., Irizarry R.A., Feinberg A.P. (2009). Large histone H3 lysine 9 dimethylated chromatin blocks distinguish differentiated from embryonic stem cells. Nat. Genet..

[21] Hirano Y., Hizume K., Kimura H., Takeyasu K., Haraguchi T., Hiraoka Y. (2012). Lamin B receptor recognizes specific modifications of histone H4 in heterochromatin formation. J. Biol. Chem..

[22] Clowney E.J., LeGros M.A., Mosley C.P., Clowney F.G., Markenskoff-Papadimitriou E.C., Myllys M., Barnea G., Larabell C.A., Lomvardas S. (2012). Nuclear aggregation of olfactory receptor genes governs their monogenic expression. Cell.

[23] Lukášová E., Kovarík A., Bacíková A., Falk M., Kozubek S. (2017). Loss of lamin B receptor is necessary to induce cellular senescence. Biochem. J..

[24] Zuleger N., Boyle S., Kelly D.A., de las Heras J.I., Lazou V., Korfali N., Batrakou D.G., Randles K.N., Morris G.E., Harrison D.J. (2013). Specific nuclear envelope transmembrane proteins can promote the location of chromosomes to and from the nuclear periphery. Genome Biol..

[25] Robson M.I., de Las Heras J.I., Czapiewski R., Lê Thành P., Booth D.G., Kelly D.A., Webb S., Kerr A.R.W., Schirmer E.C. (2016). Tissue-specific gene repositioning by muscle nuclear membrane proteins enhances repression of critical developmental genes during myogenesis. Mol. Cell.

[26] Höger T.H., Krohne G., Kleinschmidt J.A. (1991). Interaction of *Xenopus* lamins A and LII with chromatin in vitro mediated by a sequence element in the carboxyterminal domain. Exp. Cell Res..

[27] Luderus M.E., den Blaauwen J.L., de Smit O.J., Compton D.A., van Driel R. (1994). Binding of matrix attachment regions to lamin polymers involves single-stranded regions and the minor groove. Mol. Cell. Biol..

[28] Goldberg M., Harel A., Brandeis M., Rechsteiner T., Richmond T.J., Weiss A.M., Gruenbaum Y. (1999). The tail domain of lamin Dm0 binds histones H2A and H2B. Proc. Natl. Acad. Sci. USA.

[29] Shevelyov Y.Y., Lavrov S.A., Mikhaylova L.M., Nurminsky I.D., Kulathinal R.J., Egorova K.S., Rozovsky Y.M., Nurminsky D.I. (2009). The B-type lamin is required for somatic repression of testis-specific gene clusters. Proc. Natl. Acad. Sci. USA.

[30] Kohwi M., Lupton J.R., Lai S.L., Miller M.R., Doe C.Q. (2013). Developmentally regulated subnuclear genome reorganization restricts neural progenitor competence in *Drosophila*. Cell.

[31] Malhas A., Lee C.F., Sanders R., Saunders N.J., Vaux D.J. (2007). Defects in lamin B1 expression or processing affect interphase chromosome position and gene expression. J. Cell Biol..

[32] Solovei I., Kreysing M., Lanctôt C., Kösem S., Peichl L., Cremer T., Guck J., Joffe B. (2009). Nuclear architecture of rod photoreceptor cells adapts to vision in mammalian evolution. Cell.

[33] Ulianov S.V., Doronin S.A., Khrameeva E.E., Kos P.I., Luzhin A.V., Starikov S.S., Galitsyna A.A., Nenasheva V.V., Ilyin A.A., Flyamer I.M. (2019). Nuclear lamina integrity is required for proper spatial organization of chromatin in *Drosophila*. Nat. Commun..

[34] Zheng X., Hu J., Yue S., Kristiani L., Kim M., Sauria M., Taylor J., Kim Y., Zheng Y. (2018). Lamins organize the global three-dimensional genome from the nuclear periphery. Mol. Cell.

[35] Zheng X., Kim Y., Zheng Y. (2015). Identification of lamin B-regulated chromatin regions based on chromatin landscapes. Mol. Biol. Cell.

[36] Lenz-Böhme B., Wismar J., Fuchs S., Reifegerste R., Buchner E., Betz H., Schmitt B. (1997). Insertional mutation of the *Drosophila* nuclear lamin *Dm0* gene results in defective nuclear envelopes, clustering of nuclear pore complexes, and accumulation of annulate lamellae. J. Cell Biol..

[37] Liu J., Rolef Ben-Shahar T., Riemer D., Treinin M., Spann P., Weber K., Fire A., Gruenbaum Y. (2000). Essential roles for *Caenorhabditis elegans* lamin gene in nuclear organization, cell cycle progression, and spatial organization of nuclear pore complexes. Mol. Biol. Cell.

[38] Wagner N., Schmitt J., Krohne G. (2004). Two novel LEM-domain proteins are splice products of the annotated *Drosophila melanogaster* gene *CG9424* (*Bocksbeutel*). Eur. J. Cell Biol..

[39] Guo Y., Kim Y., Shimi T., Goldman R.D., Zheng Y. (2014). Concentration-dependent lamin assembly and its roles in the localization of other nuclear proteins. Mol. Biol. Cell.

[40] Kim Y., Sharov A.A., McDole K., Cheng M., Hao H., Fan C.M., Gaiano N., Ko M.S., Zheng Y. (2011). Mouse B-type lamins are required for proper organogenesis but not by embryonic stem cells. Science.

[41] Yang S.H., Chang S.Y., Yin L., Tu Y., Hu Y., Yoshinaga Y., de Jong P.J., Fong L.G., Young S.G. (2011). An absence of both lamin B1 and lamin B2 in keratinocytes has no effect on cell proliferation or the development of skin and hair. Hum. Mol. Genet..

[42] Hancock R. (2007). Packing of the polynucleosome chain in interphase chromosomes: Evidence for a contribution of crowding and entropic forces. Semin. Cell. Dev. Biol..

[43] Dixon J.R., Selvaraj S., Yue F., Kim A., Li Y., Shen Y., Hu M., Liu J.S., Ren B. (2012). Topological domains in mammalian genomes identified by analysis of chromatin interactions. Nature.

[44] Hou C., Li L., Qin Z.S., Corces V.G. (2012). Gene density, transcription, and insulators contribute to the partition of the *Drosophila* genome into physical domains. Mol. Cell.

[45] Sexton T., Yaffe E., Kenigsberg E., Bantignies F., Leblanc B., Hoichman M., Parrinello H., Tanay A., Cavalli G. (2012). Three-dimensional folding and functional organization principles of the *Drosophila* genome. Cell.

[46] Ulianov S.V., Khrameeva E.E., Gavrilov A.A., Flyamer I.M., Kos P., Mikhaleva E.A., Penin A.A., Logacheva M.D., Imakaev M.V., Chertovich A. (2016). Active chromatin and transcription play a key role in chromosome partitioning into topologically associating domains. Genome Res..

[47] Wu F., Yao J. (2013). Spatial compartmentalization at the nuclear periphery characterized by genome-wide mapping. BMC Genomics.

[48] Wu F., Yao J. (2017). Identifying novel transcriptional and epigenetic features of nuclear lamina-associated genes. Sci. Rep..

[49] Pindyurin A.V., Ilyin A.A., Ivankin A.V., Tselebrovsky M.V., Nenasheva V.V., Mikhaleva E.A., Pagie L., van Steensel B., Shevelyov Y.Y. (2018). The large fraction of heterochromatin in *Drosophila* neurons is bound by both B-type lamin and HP1a. Epigenetics Chromatin.

[50] Finlan L.E., Sproul D., Thomson I., Boyle S., Kerr E., Perry P., Ylstra B., Chubb J.R., Bickmore W.A. (2008). Recruitment to the nuclear periphery can alter expression of genes in human cells. PLoS Genet..

[51] Reddy K.L., Zullo J.M., Bertolino E., Singh H. (2008). Transcriptional repression mediated by repositioning of genes to the nuclear lamina. Nature.

[52] Dialynas G., Speese S., Budnik V., Geyer P.K., Wallrath L.L. (2010). The role of *Drosophila* Lamin C in muscle function and gene expression. Development.

[53] Wang H., Xu X., Nguyen C.M., Liu Y., Gao Y., Lin X., Daley T., Kipniss N.H., La Russa M., Qi L.S. (2018). CRISPR-mediated programmable 3D genome positioning and nuclear organization. Cell.

[54] Kind J., Pagie L., Ortabozkoyun H., Boyle S., de Vries S.S., Janssen H., Amendola M., Nolen L.D., Bickmore W.A., van Steensel B. (2013). Single-cell dynamics of genome-nuclear lamina interactions. Cell.

[55] Kind J., Pagie L., de Vries S.S., Nahidiazar L., Dey S.S., Bienko M., Zhan Y., Lajoie B., de Graaf C.A., Amendola M. (2015). Genome-wide maps of nuclear lamina interactions in single human cells. Cell.

[56] Németh A., Conesa A., Santoyo-Lopez J., Medina I., Montaner D., Péterfia B., Solovei I., Cremer T., Dopazo J., Längst G. (2010). Initial genomics of the human nucleolus. PLoS Genet..

[57] Wijchers P.J., Geeven G., Eyres M., Bergsma A.J., Janssen M., Verstegen M., Zhu Y., Schell Y., Vermeulen C., de Wit E. (2015). Characterization and dynamics of pericentromere-associated domains in mice. Genome Res..

[58] Lieberman-Aiden E., van Berkum N.L., Williams L., Imakaev M., Ragoczy T., Telling A., Amit I., Lajoie B.R., Sabo P.J., Dorschner M.O. (2009). Comprehensive mapping of long-range interactions reveals folding principles of the human genome. Science.

[59] Somech R., Shaklai S., Geller O., Amariglio N., Simon A.J., Rechavi G., Gal-Yam E.N. (2005). The nuclear-envelope protein and transcriptional repressor LAP2β interacts with HDAC3 at the nuclear periphery, and induces histone H4 deacetylation. J. Cell Sci..

[60] Holaska J.M., Wilson K.L. (2007). An emerin "proteome": Purification of distinct emerin-containing complexes from HeLa cells suggests molecular basis for diverse roles including gene regulation, mRNA splicing, signaling, mechanosensing, and nuclear architecture. Biochemistry.

[61] Dechat T., Gesson K., Foisner R. (2010). Lamina-independent lamins in the nuclear interior serve important functions. Cold Spring Harb. Symp. Quant. Biol..

[62] Gesson K., Rescheneder P., Skoruppa M.P., von Haeseler A., Dechat T., Foisner R. (2016). A-type lamins bind both hetero- and euchromatin, the latter being regulated by lamina-associated polypeptide 2 alpha. Genome Res..

[63] Tatli M., Medalia O. (2018). Insight into the functional organization of nuclear lamins in health and disease. Curr. Opin. Cell Biol..

[64] Ahmed K., Dehghani H., Rugg-Gunn P., Fussner E., Rossant J., Bazett-Jones D.P. (2010). Global chromatin architecture reflects pluripotency and lineage commitment in the early mouse embryo. PLoS ONE.

[65] Meister P., Mango S.E., Gasser S.M. (2011). Locking the genome: Nuclear organization and cell fate. Curr. Opin. Genet. Dev..

[66] Pindyurin A.V., Pagie L., Kozhevnikova E.N., van Arensbergen J., van Steensel B. (2016). Inducible DamID systems for genomic mapping of chromatin proteins in *Drosophila*. Nucleic Acids Res..

[67] Filion G.J., van Bemmel J.G., Braunschweig U., Talhout W., Kind J., Ward L.D., Brugman W., de Castro I.J., Kerkhoven R.M., Bussemaker H.J. (2010). Systematic protein location mapping reveals five principal chromatin types in *Drosophila* cells. Cell.

[68] Gigante C.M., Dibattista M., Dong F.N., Zheng X., Yue S., Young S.G., Reisert J., Zheng Y., Zhao H. (2017). Lamin B1 is required for mature neuron-specific gene expression during olfactory sensory neuron differentiation. Nat. Commun..

[69] Chen H., Zheng X., Zheng Y. (2014). Age-associated loss of lamin-B leads to systemic inflammation and gut hyperplasia. Cell.

[70] Zhu J., Adli M., Zou J.Y., Verstappen G., Coyne M., Zhang X., Durham T., Miri M., Deshpande V., De Jager P.L. (2013). Genome-wide chromatin state transitions associated with developmental and environmental cues. Cell.

[71] Ugarte F., Sousae R., Cinquin B., Martin E.W., Krietsch J., Sanchez G., Inman M., Tsang H., Warr M., Passegué E. (2015). Progressive chromatin condensation and H3K9 methylation regulate the differentiation of embryonic and hematopoietic stem cells. Stem Cell Rep..

[72] Marshall O.J., Brand A.H. (2017). Chromatin state changes during neural development revealed by in vivo cell-type specific profiling. Nat. Commun..

[73] Larson A.G., Elnatan D., Keenen M.M., Trnka M.J., Johnston J.B., Burlingame A.L., Agard D.A., Redding S., Narlikar G.J. (2017). Liquid droplet formation by HP1α suggests a role for phase separation in heterochromatin. Nature.

[74] Strom A.R., Emelyanov A.V., Mir M., Fyodorov D.V., Darzacq X., Karpen G.H. (2017). Phase separation drives heterochromatin domain formation. Nature.

[75] Banani S.F., Lee H.O., Hyman A.A., Rosen M.K. (2017). Biomolecular condensates: Organizers of cellular biochemistry. Nat. Rev. Mol. Cell. Biol..

[76] Ma Y., Buttitta L. (2017). Chromatin organization changes during the establishment and maintenance of the postmitotic state. Epigenet. Chromatin.

[77] Lee Y.C.G., Karpen G.H. (2017). Pervasive epigenetic effects of *Drosophila* euchromatic transposable elements impact their evolution. eLife.

[78] Criscione S.W., Teo Y.V., Neretti N. (2016). The chromatin landscape of cellular senescence. Trends Genet..

[79] Narita M., Nũnez S., Heard E., Narita M., Lin A.W., Hearn S.A., Spector D.L., Hannon G.J., Lowe S.W. (2003). Rb-mediated heterochromatin formation and silencing of E2F target genes during cellular senescence. Cell.

[80] Zhang R., Chen W., Adams P.D. (2007). Molecular dissection of formation of senescence-associated heterochromatin foci. Mol. Cell. Biol..

[81] Chandra T., Kirschner K., Thuret J.Y., Pope B.D., Ryba T., Newman S., Ahmed K., Samarajiwa S.A., Salama R., Carroll T. (2012). Independence of repressive histone marks and chromatin compaction during senescent heterochromatic layer formation. Mol. Cell.

[82] Shimi T., Butin-Israeli V., Adam S.A., Hamanaka R.B., Goldman A.E., Lucas C.A., Shumaker D.K., Kosak S.T., Chandel N.S., Goldman R.D. (2011). The role of nuclear lamin B1 in cell proliferation and senescence. Genes Dev..

[83] Lenain C., Gusyatiner O., Douma S., van den Broek B., Peeper D.S. (2015). Autophagy-mediated degradation of nuclear envelope proteins during oncogene-induced senescence. Carcinogenesis.

[84] Dreesen O., Chojnowski A., Ong P.F., Zhao T.Y., Common J.E., Lunny D., Lane E.B., Lee S.J., Vardy L.A., Stewart C.L. (2013). Lamin B1 fluctuations have differential effects on cellular proliferation and senescence. J. Cell Biol..

[85] Lenain C., de Graaf C.A., Pagie L., Visser N.L., de Haas M., de Vries S.S., Peric-Hupkes D., van Steensel B., Peeper D.S. (2017). Massive reshaping of genome-nuclear lamina interactions during oncogene-induced senescence. Genome Res..

[86] Chandra T., Ewels P.A., Schoenfelder S., Furlan-Magaril M., Wingett S.W., Kirschner K., Thuret J.Y., Andrews S., Fraser P., Reik W. (2015). Global reorganization of the nuclear landscape in senescent cells. Cell Rep..

[87] De Cecco M., Criscione S.W., Peckham E.J., Hillenmeyer S., Hamm E.A., Manivannan J., Peterson A.L., Kreiling J.A., Neretti N., Sedivy J.M. (2013). Genomes of replicatively senescent cells undergo global epigenetic changes leading to gene silencing and activation of transposable elements. Aging Cell.

[88] Criscione S.W., De Cecco M., Siranosian B., Zhang Y., Kreiling J.A., Sedivy J.M., Neretti N. (2016). Reorganization of chromosome architecture in replicative cellular senescence. Sci. Adv..

[89] Eriksson M., Brown W.T., Gordon L.B., Glynn M.W., Singer J., Scott L., Erdos M.R., Robbins C.M., Moses T.Y., Berglund P. (2003). Recurrent *de novo* point mutations in lamin A cause Hutchinson-Gilford progeria syndrome. Nature.

[90] Goldman R.D., Shumaker D.K., Erdos M.R., Eriksson M., Goldman A.E., Gordon L.B., Gruenbaum Y., Khuon S., Mendez M., Varga R. (2004). Accumulation of mutant lamin A causes progressive changes in nuclear architecture in Hutchinson-Gilford progeria syndrome. Proc. Natl. Acad. Sci. USA.

[91] Shumaker D.K., Dechat T., Kohlmaier A., Adam S.A., Bozovsky M.R., Erdos M.R., Eriksson M., Goldman A.E., Khuon S., Collins F.S. (2006). Mutant nuclear lamin A leads to progressive alterations of epigenetic control in premature aging. Proc. Natl. Acad. Sci. USA.

[92] McCord R.P., Nazario-Toole A., Zhang H., Chines P.S., Zhan Y., Erdos M.R., Collins F.S., Dekker J., Cao K. (2013). Correlated alterations in genome organization, histone methylation, and DNA-lamin A/C interactions in Hutchinson-Gilford progeria syndrome. Genome Res..

[93] Solovei I., Thanisch K., Feodorova Y. (2016). How to rule the nucleus: Divide *et impera*. Curr. Opin. Cell Biol..

[94] Mo A., Luo C., Davis F.P., Mukamel E.A., Henry G.L., Nery J.R., Urich M.A., Picard S., Lister R., Eddy S.R. (2016). Epigenomic landscapes of retinal rods and cones. eLife.

[95] Hughes A.E., Enright J.M., Myers C.A., Shen S.Q., Corbo J.C. (2017). Cell type-specific epigenomic analysis reveals a uniquely closed chromatin architecture in mouse rod photoreceptors. Sci. Rep..

[96] Falk M., Feodorova Y., Naumova N., Imakaev M., Lajoie B.R., Leonhardt H., Joffe B., Dekker J., Fudenberg G., Solovei I. (2018). Heterochromatin drives organization of conventional and inverted nuclei. bioRxiv.

[97] Bannister A.J., Zegerman P., Partridge J.F., Miska E.A., Thomas J.O., Allshire R.C., Kouzarides T. (2001). Selective recognition of methylated lysine 9 on histone H3 by the HP1 chromo domain. Nature.

[98] Lachner M., O’Carroll D., Rea S., Mechtler K., Jenuwein T. (2001). Methylation of histone H3 lysine 9 creates a binding site for HP1 proteins. Nature.

[99] Harr J.C., Luperchio T.R., Wong X., Cohen E., Wheelan S.J., Reddy K.L. (2015). Directed targeting of chromatin to the nuclear lamina is mediated by chromatin state and A-type lamins. J. Cell Biol..

[100] Kharchenko P.V., Alekseyenko A.A., Schwartz Y.B., Minoda A., Riddle N.C., Ernst J., Sabo P.J., Larschan E., Gorchakov A.A., Gu T. (2011). Comprehensive analysis of the chromatin landscape in *Drosophila melanogaster*. Nature.

[101] Kubben N., Voncken J.W., Konings G., van Weeghel M., van den Hoogenhof M.M., Gijbels M., van Erk A., Schoonderwoerd K., van den Bosch B., Dahlmans V. (2011). Post-natal myogenic and adipogenic developmental: Defects and metabolic impairment upon loss of A-type lamins. Nucleus.

